# Asymmetric and Spatial Non-Stationary Effects of Particulate Air Pollution on Urban Housing Prices in Chinese Cities

**DOI:** 10.3390/ijerph17207443

**Published:** 2020-10-13

**Authors:** Biao Sun, Shan Yang

**Affiliations:** 1 School of Geographic Science, Nanjing Normal University, Nanjing 210023, China; sunbiaoyf@163.com; 2 Jiangsu Center for Collaborative Innovation in Geographical Information Resource Development and Application, Nanjing 210023, China

**Keywords:** urban housing prices, particulate matter air pollution, quantile regression, geographically weighted quantile regression, prefecture-level cities, China

## Abstract

Fine particulate matter(PM2.5) pollution will affect people’s well-being and cause economic losses. It is of great value to study the impact of PM2.5 on the real estate market. While previous studies have examined the effects of PM2.5 pollution on urban housing prices, there has been little in-depth research on these effects, which are spatially heterogeneous at different conditional quantiles. To address this issue, this study employs quantile regression (QR) and geographically weighted quantile regression (GWQR) models to obtain a full account of asymmetric and spatial non-stationary effects of PM2.5 pollution on urban housing prices through 286 Chinese prefecture-level cities for 2005–2013. Considerable differences in the data distributions and spatial characteristics of PM2.5 pollution and urban housing prices are found, indicating the presence of asymmetric and spatial non-stationary effects. The quantile regression results show that the negative influences of PM2.5 pollution on urban housing prices are stronger at higher quantiles and become more pronounced with time. Furthermore, the spatial relationship between PM2.5 pollution and urban housing prices is spatial non-stationary at most quantiles for the study period. A negative correlation gradually dominates in most of the study areas. At higher quantiles, PM2.5 pollution is always negatively correlated with urban housing prices in eastern coastal areas and is stable over time. Based on these findings, we call for more targeted approaches to regional real estate development and environmental protection policies.

## 1. Introduction

Through China’s rapid urbanization over the past 40 years, more than 600 million people have migrated from rural areas to cities, creating considerable demand for urban housing, thus promoting urban economic and real estate market development [1]. However, population agglomeration and rapid social and economic development have also led to increased demand for resources and energy [2]. To ensure the normal operation and rapid development of cities, large-scale industrial production, fossil fuel resource consumption, and energy extraction are required. Chemical plant production and the burning of fossil fuels create serious air pollution problems such as those related to PM2.5 pollution [3,4]. PM2.5 pollution compromises not only productivity but also residents’ welfare [3]. In China, few cities meet standards of air quality guideline established by the World Health Organization (WHO). Even the cleanest city of Sanya reports double the levels set by the WHO with annual average PM2.5 concentrations of 21 µg/m^3^ in 2013 [3].

A highly polluted environment is generally considered to be harmful to human health [5,6,7,8,9]. In particular, fine particulate matter, which is known as PM2.5 widely, is a pollutant that is very harmful with extensive influences [10,11,12]. Long-term exposure to high concentrations of PM2.5 environment may cause serious health impacts such as cardiovascular and respiratory disease, lung cancer, and cerebrovascular disease [7,13,14]. The possibility and risk of cancer is increased by the influence of polycyclic aromatic hydrocarbons and other substances in the particles [15,16]. An estimated 4.5 billion people exhibited health risks due to the exposure to PM2.5 concentrations in 2016, increasing the mortality and economic costs [17]. A study of 338 Chinese prefecture-level cities showed that mortality attributable to PM2.5 reached the million level [18]. Moreover, the economic loss caused by PM2.5 exposure was 101.39 billion dollars, accounting for 0.91% of the GDP in 2016 [18]. On the basis of these facts, it can be inferred that there would be certain effects of PM2.5 pollution on urban housing prices. With improvements in living standards, people are paying more attention to living quality when considering where to settle. Residents’ willingness to invest in highly polluted areas is affected, naturally depressing the housing prices. Several studies have confirmed that housing prices are influenced by air pollution negatively [3,19,20,21]. According to the research used the instrumental variable regression method on the panel data of Chinese cities from 2004 to 2013, it was accurately estimated that every increase of 1 µg/m^3^ in PM2.5 concentration will lead to a decrease of 46 RMB/m^2^ in housing prices [21]. When the robustness test was carried out in the form of logarithm of variables, every 1% increase in PM2.5 concentration is associated with 0.43% decrease of housing values [21]. Zheng, Cao, Kahn, and Sun (2014) found that a 10 % increase in imported neighbor pollution is associated with a 0.76 % decrease in housing prices on basis of a sample of 85 Chinese cities and detected that cross-boundary flows drives this capitalization growth [22]. In addition, Hao and Zheng’s (2017) analysis showed that housing prices are negatively influenced by pollution sources such as sulfur dioxide emissions, industrial soot emissions, and so forth [23]. The magnitude of this influence depends on levels of economic development, while housing values in cities with high per capita GDP are less influenced by environmental pollution [23].

The impacts of PM2.5 pollution on cities with different housing prices should be various. For instance, as metropolises provide numerous employment opportunities [24], they attract individuals who do not consider air pollution levels into their decisions on where to settle, resulting in an overestimation of negative influences of air pollution on housing values. Chen and Chen (2017) revealed that negative influences of PM2.5 pollution vary considerably across cities with magnitudes declining sharply from high- to low-ranking cities [21]. Specifically, housing values of first- and second-tier cities decrease over four times more than those of third-tier cities. While the impacts of different housing prices were successfully measured, the study classified cities and directly applied ordinary least squares (OLS) regression, in turn truncating the dependent variable and creating estimation errors. Therefore, for measuring impacts of PM2.5 pollution on housing prices in cities of different levels, a more accurate estimation model is needed. Quantile regression model can effectively cope with this problem [25,26], and can reveal the asymmetric effects of PM2.5 pollution on urban housing prices at different quantiles.

In addition, there are spatial differences in the impacts of air pollution on regions [27]. Scholars have proved the existence of spatial heterogeneity in related research [28,29,30]. Spatial heterogeneity and less pronounced negative influences of air pollution on housing values in the southeastern area of the study region were found through a case study of Salt Lake County, Utah [31]. China is a vast country with pronounced regional differences, and spatial inequalities in housing markets have been shaped in different cities due to uneven distributions of large and small cities [24]. Hence, spatial heterogeneity ought to be considered when studying the influences of PM2.5 pollution on urban housing values. Moving window regression (MWR) and geographically weighted regression (GWR) approaches have been developed and conformed to be more accurate than OLS regression [32,33,34,35]. Moreover, with respect to prediction accuracy and explanatory power, GWR is superior to MWR [35].

The GWR model is an inherent mean regression model and is not robust to outliers on account of using the least squares criterion [36], therefore some scholars have considered the use of spatial quantile methods to address this problem [36,37,38]. Hanllin’s local linear spatial quantile regression approach provided much richer information [37]. On the basis of the least absolute deviation, Zhang and Mei (2011) proposed a geographically weighted median regression method [36]. Chen, Deng, Yang, and Matthews (2012) considered a more generalized GWQR model and introduced a solution for analyzing spatial non-stationarity in the specified quantile estimation coefficient [38]. Wang, Xu, and Yan (2018) integrated lasso regression into GWQR to alleviate collinearity complications following the framework of Chen et al. (2012) [39]. Using traditional QR and GWQR methods to model incident rates of children’s lead poisoning, Zhen, Cao, Shao, and Zhang (2018) identified ‘high risk’ locations and neighborhoods of inner city Syracuse and found that the GWQR model can provide more comprehensive information for policymakers [40].

Scholars have examined asymmetry and spatial non-stationarity in housing values by using QR and GWR analysis frameworks, respectively. To our knowledge, no research has considered both asymmetric and spatial non-stationary effects of PM2.5 pollution on housing prices in Chinese cities. To bridge these gaps, this study provides empirical evidence of a relationship between PM2.5 pollution and housing prices. The following research problems will be explored in our work: (1) Do PM2.5 concentrations reduce housing prices? (2) Are the influences of PM2.5 pollution on housing prices asymmetric at different quantiles? (3) Are these influences spatial non-stationary? First, we use a traditional quantile regression model to measure how differences between prefecture-level cities at different housing price quantiles are affected by PM2.5 concentrations over three cross-section time periods of 2005, 2009, and 2013. Second, we use the GWQR model to further refine our study and to assess how spatial heterogeneity in PM2.5 pollution impacts housing prices at different quantiles. Unlike past works, this study comprehensively considers the impact of PM2.5 pollution on urban housing prices at different quantiles. To our knowledge, GWQR is applied to measure the spatial non-stationary influences of PM2.5 pollution on urban housing prices for the first time. Based on accurate assessment of these influences, we provide some policy implications about the real estate development and regional environmental protection from the perspectives of housing values and location differences.

## 2. Materials and Methods

### 2.1. Theoretical Basis of Housing Prices Researches

Research on housing prices has been carried out at multiple scales. At the local scale, housing prices refer to the prices of individual houses, and scholars regard houses as composite commodities [41,42], with housing prices defined as the sum of consumers’ utilities [43]. Based on the hedonic price model developed by Rosen [44], houses’ inherent, neighborhood, and location attributes are capitalized into housing prices [45,46,47,48]. Unlike local-scale research, macro-scale studies offer overall reflections of regional real estate markets and can provide a basis for differentiated formulations of housing prices control and real estate market development policies.

At the city scale, housing prices are calculated as the average household sales price for a city [49,50,51,52]. In early research, based on the theory of market supply and demand equilibrium, scholars usually analyzed the influencing factors of housing price changes through simultaneous equations model [53]. Some scholars regard housing as a durable good, and there are obvious time lag effects between supply and demand. Simple supply and demand models are not enough to explain the formation mechanism of housing prices, and the modified dynamic stock-flow model is proposed to reflect the changes of housing market [54]. More follow-up studies found that influencing factors of housing prices are very complex due to the dual attributes of consumer goods and investment goods. Especially when it comes to China’s housing prices, which have been continuously pushed up by capital in pursuit of profit in recent years, and seem to have deviated from the rational framework of the consumer goods market, the traditional market supply and demand theory of housing prices has become increasingly difficult to work with. According to the existing literature, the influencing factors of urban housing prices include at least the macro-level economic situation and policies [55,56], the city’s economic level [57], the income level of residents [58], the population size and agglomeration capacity [59], the quality of public services [60,61], the air environmental health, and so forth [3,21]. These factors also show spatial heterogeneity. Although there is no unified theory that can solve the influencing factors of housing prices, we can find that housing prices are closely related to the abundance of resources such as urban economy, population, public services and environmental livability. Therefore, we believe that housing prices can also be capitalized as an expression of these resource capacity differences at the macro level.

### 2.2. Study Area

**Figure 1:**
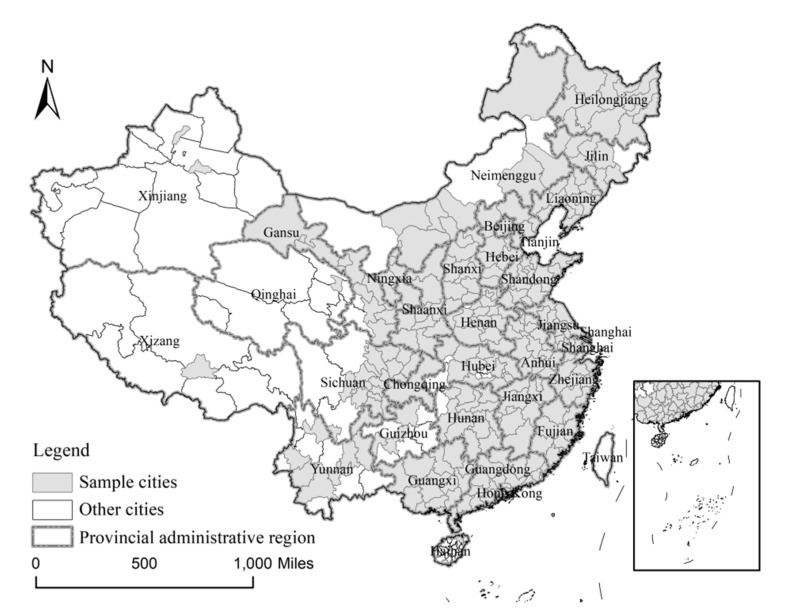
Study area.

We use the prefecture as our research unit. Prefectures in China are second-level administrative divisions ranked between provinces and counties. In studying prefectures, we obtain a larger sample than we would have at the provincial scale and more detailed statistical data than we would have at the county scale. Thus, the sizes of prefectures render them an appropriate unit of analysis [62]. We select 286 prefecture-level cities and above with relatively complete information for the study period. Figure 1 maps the locations of the sample cities and boundary of the provincial administrative region. These samples have complete information on administrative jurisdiction and detailed statistical data [63], and they are distributed across most of China, which are thus nationally representative [21].

### 2.3. Data and Variables

For the purpose of investigating the influences of PM2.5 pollution on urban housing prices, PM2.5 pollution is used as our core independent variable and urban pollution level is measured by PM2.5 concentrations. According to the existing literature [3,21], we also employ other forms of air pollution, demographic and socioeconomic conditions, and urban public facilities as control variables. Variable definitions and descriptive statistics are reported in Table 1. To eliminate heteroscedasticity in the data and ensure stability in the regression, all variables are logarithmic. Therefore, the estimates of variables can be explained as elasticities [64].

#### 2.3.1. Urban Housing Prices

We take urban housing prices as our dependent variable, and extract data from the China Statistical Yearbook for Regional Economies. We calculate average housing prices based on total housing sales and the total housing sales area of each prefecture-level city. The average prices of newly built commercial housing can approximately represent housing prices in a city because of their major market share (over 70%) of housing transactions in China [22]. Since the China Statistical Yearbook for Regional Economies was only updated to 2013, we collected the data on Chinese prefecture-level housing prices from 2005 to 2013.

#### 2.3.2. PM2.5 Pollution

China did not release PM2.5 data from monitoring stations until 2012 [65]; thus, research on PM2.5 concentrations has been limited to some extent [21]. However, the Socioeconomic Data and Applications Centre (SEDAC) has published global PM2.5 grid datasets (0.01 degrees) for 1998 to 2017 that are accurate and reflect changes in particulate air pollution in China as shown by relevant studies [66,67]. For the purposes of our analysis, the raster data are transformed into the annual average PM2.5 concentrations for prefecture-level cities through ArcGIS software (ESRI, Redlands, CA, USA).

#### 2.3.3. Other Variables

We also control other factors potentially related to urban housing prices in Table 1 [21], which helps us minimize estimation errors and endogeneity problems caused by missing variables. These data are taken from the China City Statistical Yearbook.

### 2.4. Methodology

In our study, the asymmetric effects of PM2.5 pollution on urban housing prices are explored by quantile regression. Then, geographically weighted quantile regression, a newly developed method, is used to measure spatial non-stationary effects of the response variable in geographic regions at different quantiles.

#### 2.4.1. Quantile Regression

In the traditional quantile regression model, a conditional quantile of dependent variable is specified as a linear function of explanatory variables, which is given by [68]:

(1) $$Y_{i}=X_{i}^{t}\beta^{\tau }+\varepsilon_{i}$$

(2) $$q_{\tau }\left(X_{i}\right)=X_{i}^{t}\beta^{\tau }$$

where $Y_{i}$ is the dependent variable, $X_{i}$ refers to the matrix of explanatory variables, $\varepsilon_{i}$ is the error term whose conditional quantile distribution equals zero, and unknown sets. $q_{\tau }\left(X_{i}\right)$ is the τth quantile of $Y_{i}$ conditional on $X_{i}$. QR coefficient $\beta^{\tau }$ is estimated by minimizing the following loss function [68]:

(3) $$\min\overset{n}{\underset{i=1}{\sum }}\rho_{\tau }\left(Y_{i}-X_{i}^{t}\beta^{\tau }\right)$$

where $\rho_{\tau }\left(z\right)=z(\tau -I[z<0])$ is a V-shaped piecewise linear loss function and I(·) is the indicator function [68].

The following equation is the quantile regression model used to obtain the comprehensive results in this study:

(4) $$\begin{array}{ll} \ln\left(HP\right)= & \beta_{0}^{\tau }+\beta_{1}^{\tau }\ln\left(\mathrm{PM}2.5\right)+\beta_{2}^{\tau }\ln\left(\mathrm{SO}2\right)+\beta_{3}^{\tau }\ln\left(\mathrm{Soot}\right)+\beta_{4}^{\tau }\ln\left(\mathrm{PDen}\right)+\beta_{5}^{\tau }\ln\left(\mathrm{PGDP}\right)\\ & +\beta_{6}^{\tau }\ln(\mathrm{Wage})+\beta_{7}^{\tau }\ln\left(\mathrm{Ind}\_\mathrm{Str}\right)+\beta_{8}^{\tau }\ln\left(\mathrm{PBu}\right)+\beta_{9}^{\tau }\ln\left(\mathrm{PBo}\right)+\beta_{10}^{\tau }\ln\left(\mathrm{PDoc}\right)\\ & +\beta_{11}^{\tau }\ln\left(\mathrm{PRoad}\right)+\beta_{12}^{\tau }\ln(\mathrm{Int})+\beta_{13}^{\tau }\ln\left(\mathrm{PTea}\right)+\varepsilon \end{array}$$

The detailed information of all variables is shown in Table 1.

#### 2.4.2. Geographically Weighted Quantile Regression

We extend GWR model to a quantile regression setting [38], and the following equations are estimated to study spatial heterogeneity in the regression relationship [38,39]:

(5) $$Y_{i}=X_{i}^{t}\beta^{\tau }\left(u_{i},v_{i}\right)+\varepsilon_{i}^{\tau }=\;\beta_{0}^{\tau }\left(u_{i},v_{i}\right)+\overset{p}{\underset{k=1}{\sum }}X_{ik}\beta_{k}^{\tau }\left(u_{i},v_{i}\right)+\varepsilon_{i}^{\tau }$$

(6) $$q_{\tau }\left(X_{i},u_{i},v_{i}\right)=X_{i}^{t}\beta^{\tau }\left(u_{i},v_{i}\right)=\beta_{0}^{\tau }\left(u_{i},v_{i}\right)+\overset{p}{\underset{k=1}{\sum }}X_{ik}\beta_{k}^{\tau }\left(u_{i},v_{i}\right)$$

where $\varepsilon_{i}^{\tau }$ is the random error with the conditional τth quantile being equal to zero, and $q_{\tau }\left(X_{i},u_{i},v_{i}\right)$ is the τth conditional quantile function of $Y_{i}$ given observation vector $X_{i}$ for location *i* with coordinates $\left(u_{i},v_{i}\right)$. We assume that coefficient vector $\beta^{\tau }\left(u,v\right)$ has second continuous partial derivatives in regard to *u* and *v*. $\beta_{k}^{\tau }\left(u,v\right)$ can be approximated by the linear function for (u,v) in a neighborhood of $\left(u_{0},v_{0}\right)$ in Equation (6) [38,39].

(7) $$\beta_{k}^{\tau }\left(u,v\right)\approx \beta_{k}^{\tau }\left(u_{0},v_{0}\right)+\beta_{k}^{\tau \left(u\right)}\left(u_{0},v_{0}\right)\left(u-u_{0}\right)+\beta_{k}^{\tau \left(v\right)}\left(u_{0},v_{0}\right)\left(v-v_{0}\right)$$

For a focal location $\left(u_{t},v_{t}\right)$, let $d_{it}=\|\left(u_{i},v_{i}\right)-\left(u_{t},v_{t}\right)\|$, where ‖·‖ is the usual Euclidean norm. Via minimizing the geographically weighted loss function, GWQR coefficients for the τth quantile can be obtained in Equation (7) [38,39]:

(8) $$\min\overset{n}{\underset{i=1}{\sum }}\rho_{\tau }\;\{Y_{i}-X_{i}^{t}\left[\beta^{\tau }\left(u_{t},v_{t}\right)+\beta^{\tau \left(u\right)}\left(u_{t},v_{t}\right)\left(u_{t}-u_{i}\right)+\beta^{\tau \left(v\right)}\left(u_{t},v_{t}\right)\left(v_{t}-v_{i}\right)\right]\}K_{h}\left(d_{it}\right)$$

where $\rho_{\tau }\left(z\right)$ is the V-shaped piecewise linear loss function, and $K_{h}\left(\cdot \right)$ is the Gaussian kernel function with bandwidth h, which represents the geographical weight assigned locally to observation $\left(X_{i},Y_{i}\right)$ and depends upon the distanced $d_{it}$ between the given location $\left(u_{0},v_{0}\right)$ and the τth designed location $\left(u_{i},v_{i}\right)$ [38]. We use a cross validation (CV) method to determine the optimal bandwidth. To examine spatial non-stationary effects of PM2.5 pollution on urban housing prices, we employ the approach adopted in Chen et al. (2012) [38]. We compare the interquartile range (IQR) of local coefficient estimates computed by GWQR to the standard error of global estimates derived with a traditional QR at a specified quantile. When IQR is twice as large as the standard error, this indicates that spatial non-stationary effects exist in the relationship between urban housing prices and PM2.5 pollution.

The GWQR model for location *i* at the τth quantile in this study is set as follows:

(9) $$\begin{array}{ll} \ln(HP_{i})= & \beta_{0}^{\tau }\left(u_{i},v_{i}\right)+\beta_{1}^{\tau }\left(u_{i},v_{i}\right)\ln\left(\mathrm{PM}2.5\right)+\beta_{2}^{\tau }\left(u_{i},v_{i}\right)\ln\left(\mathrm{SO}2\right)+\beta_{3}^{\tau }\left(u_{i},v_{i}\right)\ln\left(\mathrm{Soot}\right)\\ & +\beta_{4}^{\tau }\left(u_{i},v_{i}\right)\ln\left(\mathrm{PDen}\right)+\beta_{5}^{\tau }\left(u_{i},v_{i}\right)\ln\left(\mathrm{PGDP}\right)+\beta_{6}^{\tau }\left(u_{i},v_{i}\right)\ln\left(\mathrm{Wage}\right)\\ & +\beta_{7}^{\tau }\left(u_{i},v_{i}\right)\ln\left(\mathrm{Ind}\_\mathrm{Str}\right)+\beta_{8}^{\tau }\left(u_{i},v_{i}\right)\ln\left(\mathrm{PBu}\right)+\beta_{9}^{\tau }\left(u_{i},v_{i}\right)\ln\left(\mathrm{PBo}\right)\\ & +\beta_{10}^{\tau }\left(u_{i},v_{i}\right)\ln\left(\mathrm{PDoc}\right)+\beta_{11}^{\tau }\left(u_{i},v_{i}\right)\ln\left(\mathrm{PRoad}\right)+\beta_{12}^{\tau }\left(u_{i},v_{i}\right)\ln\left(\mathrm{Int}\right)\\ & +\beta_{13}^{\tau }\left(u_{i},v_{i}\right)\ln\left(\mathrm{PTea}\right)+\varepsilon_{i} \end{array}$$

The detailed information of all variables is shown in Table 1.

## 3. Results and Discussion

### 3.1. Distribution and Spatial Characteristics

**Figure 2:**
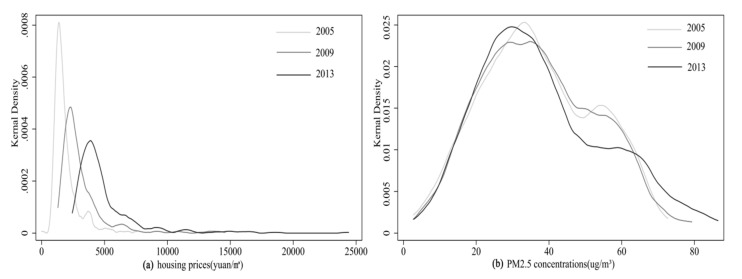
Kernel density estimate (KDE) of housing prices and PM2.5 concentrations. (**a**) Housing prices (yuan/m^2^). (**b**) PM2.5 concentrations (µg/m^3^).

From the kernel density estimate (KDE) curves (Figure 2), the distributions of urban housing prices and PM2.5 concentrations are significantly different. The distributions of housing prices are clearly asymmetric across the three time sections, and they all show a positively skewed distribution. In the time series, mode values are decreasing, showing that the distribution of the low housing price interval has become more uniform, and a shift to the right of the average value median values indicates a continuous rise in urban housing prices. The KDE curves of PM2.5 concentrations show similarities across the three time sections, which are close to forming a normal distribution. It can be concluded that the response law between them is complex and asymmetric through comparing the KDE curves.

**Figure 3:**
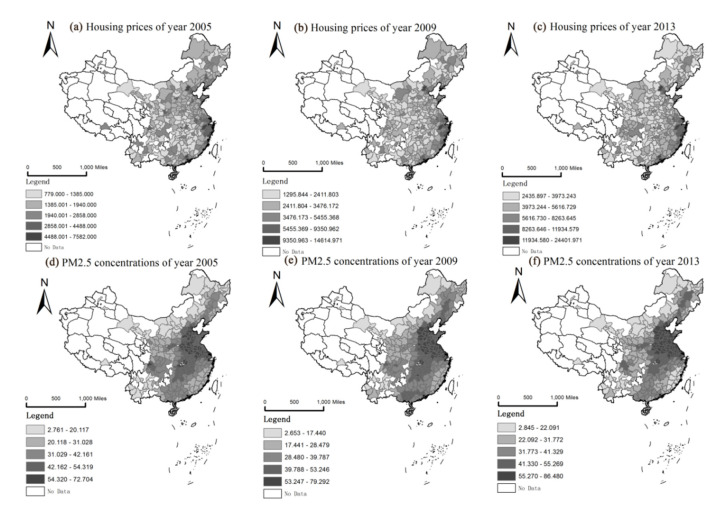
Spatial distribution of housing prices and PM2.5 concentrations from 2005 to 2013. (**a**–**c**) represent the distribution of housing prices in 2005, 2009 and 2013 respectively; (**d**–**f**) represent the distribution of PM2.5 concentrations in 2005, 2009 and 2013 respectively.

Figure 3 shows the spatial differentiation of housing prices and PM2.5 concentrations. We use the natural breaks method to divide them into 5 levels. Urban housing prices in the eastern region are higher than those in central and western regions. High-value regions are mainly concentrated in the urban agglomerations of Beijing, Shanghai, Guangzhou, and Shenzhen, and the housing prices spatial distribution remains stable in time series. However, the urban PM2.5 concentrations spatial distribution is considerably different from that of housing prices. It is clear that levels in central eastern China are significantly higher than those in peripheral areas, and the Beijing-Tianjin-Hebei urban agglomeration is a high-concentration cluster area over the three time periods. Both of them show upward trends over the time series.

**Figure 4:**
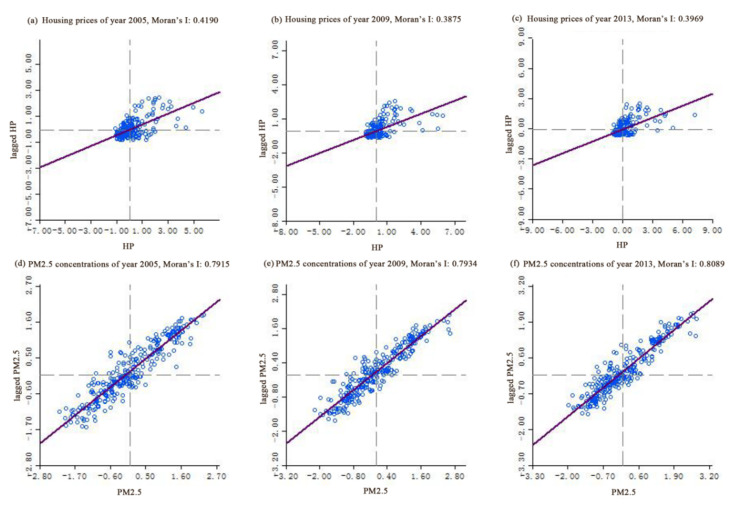
Moran’s I scatter of housing prices and PM2.5 concentrations from 2005 to 2013. (**a**–**c**) represent the Moran scatter of housing prices in 2005, 2009 and 2013 respectively; (**d**–**f**) represent the Moran scatter of PM2.5 concentrations in 2005, 2009 and 2013 respectively.

Moran scatter plot is applied to illustrate the spatial non-stationary characteristics, and the Moran’s I value is represented by the slope of the fitted line of the scatter plot (Figure 4). Moran’s I values of housing prices fluctuated around roughly 0.4 over the three time sections, indicating that urban housing prices are spatially dependent. Moran’s I values of PM2.5 concentrations are approximately valued at 0.8 and are thus twice those of housing prices. It is apparent that levels of spatial dependence are much higher than housing prices. From comparisons of spatial distribution characteristics of PM2.5 concentrations and housing prices, we can infer that spatial non-stationary effects exist between them.

### 3.2. Quantile Regression Results

The OLS and quantile regression results for 2013 are presented in Table 2. OLS results are shown in column 1 for comparative purposes. *R*^2^ is 0.7899, which indicates the good explanatory power of OLS regression model. Most of the variables are significant at the 10% level. The OLS results show that PM2.5 pollution levels are significant at the 1% level, which indicates that it can reduce housing prices. Specifically, the coefficient for PM2.5 pollution is −0.1986, indicating that every 1% increase in PM2.5 concentrations will lead to a 0.1986% decrease in housing prices.

Table 2 reports quantile regression results for the 5th, 25th, 50th, 75th, and 95th quantiles, and the pseudo *R*^2^ of selected quantiles ranges from 0.3749 to 0.7211. Moreover, *R*^2^ increases from the lower quantiles to the upper quantiles, meaning that fitting effects at the upper quantiles are better. The coefficients of PM2.5 pollution for all 5 quantile estimates are negative, and the absolute values of the upper quantiles’ regression coefficients are significantly greater than those of the lower quantile. This is the case because real estate markets with high housing prices are more speculative, leaving them more easily affected by external factors such as PM2.5 pollution. High-income individuals with investment intentions may prefer to pay for clean air. Specifically, the coefficients for the 75th and 95th quantiles are 0.2697 and 0.2480, respectively, which are more than 6 times that of the 5th quantile and 1.5 times that of the 25th quantile, echoing the results of Chen’s research on first-, second-, third-, and fourth-tier cities [21]. The 50th quantile coefficient is similar to the OLS regression coefficient, indicating that the average impact of PM2.5 pollution on housing prices approximates the median for 2013. Regarding our other air pollution variables, the coefficients of SO_2_ and soot in most quantiles are not significant, and their effects on housing prices are relatively stable in different quantiles, fluctuating around the OLS regression coefficient. PM2.5 pollution is also identified as the main form of air pollution affecting housing prices.

In addition, the OLS estimates of other variables are all significantly negative, indicating the negative impact of air pollution on housing prices (Table 2). Specifically, for every 1% increase of SO_2_ and Soot pollution, the housing prices are associated with the decrease of 0.0279% and 0.0245%, respectively. Compared with PM2.5 pollution, there is a certain gap in their impact on housing prices, which also reflects the seriousness of PM2.5 pollution. In the five selected quantiles, they are negatively correlated with housing prices (Although most of them are not significant). The coefficients of SO_2_ remained relatively stable at different quantiles, while Soot showed an upward trend. The higher housing price of a city, the greater the negative effect of Soot pollution. Similar to PM2.5 pollution, its effects on housing price are asymmetric.

Demographic and socioeconomic variables are positively correlated with housing prices (Table 2), and OLS and QR estimates of them are mostly significantly positive. The higher population density provides more housing supply, which promotes housing prices. In addition, the value-added effect of population density is also increasing in the cities with high housing prices, indicating that the land is scarcer. Similarly, higher per capita GDP and higher wage play important roles on the growth of housing prices, and Wage variable is more significant in promoting the housing prices. It is obvious that higher wage is usually the reason for attracting the flow of people, which leads to higher housing prices. The coefficients of them are bigger at higher quintiles, which is corresponding with the fact that there are the high housing price to income ratios in superstar cities [69]. The industrial structure is also one of the important factors affecting the housing prices. The cities with high-end industries have stronger innovation ability and talent attraction, which naturally leads to higher housing prices.

From the results in Table 2, only the PBo and Int variables are significantly correlated with housing prices among the variables of urban public facilities, and both of them are positive. Different from the results using panel data regression [21], the coefficients of other variables are mostly small and insignificant in the cross-section regression for 2013. It could be inferred that the value of urban public facilities would be highlighted in the long-term sequence, which also reflects that the steady increase in urban housing prices is also inseparable from the upgrade of urban public facilities.

To observe effects of PM2.5 pollution on housing prices in time series, we calculate the datasets for 2005 and 2009 to draw comparisons. The results are shown in Table 3. OLS estimates are reported in the first column. The *R*^2^ values exceed 0.75, indicating that they both have good explanatory power. The coefficients of PM2.5 pollution for 2005, 2009, and 2013 are negative, this coefficient is not significant for 2005 and it is significant at the 5% level in 2009. Relative to the absolute values of the coefficients across the three time sections, the effects of PM2.5 pollution on housing prices continuously increase. The coefficient in 2013 is nearly three times the value in 2005 (−0.0661), potentially reflecting increasing concern for the impacts of air pollution on life quality.

Columns 2–6 of Table 3 report quantile estimation results of the same quantiles examined in Table 2. Table 3 shows that PM2.5 pollution is negatively correlated with housing prices at most quantiles but only significant at the 75th quantile in 2005. The value of the coefficient is −0.1447, which is higher than that at the lower quantile, and the OLS coefficient (−0.0661) is closer to the 25th quantile (−0.0834). PM2.5 pollution has negative effects on housing prices at all quantiles in 2009 with a significance level of 1% at the 75th and 95th quantiles, and coefficient values increase from the lower to upper quantiles. The value quantile reaches −0.2571 at the 95th, which is almost 5 times that of the 5th quantile. The OLS regression coefficient value (−0.1249) falls between the 50th (−0.0751) and 75th (−0.1642) quantiles.

In summary, the impacts of PM2.5 pollution on housing prices increases in our time series, echoing Chen and Chen’s (2017) results [21]. In addition, the range of PM2.5 pollution effects is expanding, at first only affecting high quantile housing price cities and then gradually affecting cities at various quantiles, and impacts at upper quantiles are significantly greater than those that low quantiles. Improvements in living standards have increased demand for better living conditions, and more attention is thus being paid to environmental hygiene and well-being. As real estate development is no longer pursued at the expense of environment health, the real estate market is exhibiting more sensitivity to air pollution.

### 3.3. Geographically Weighted Quantile Regression Results

**Figure 5:**
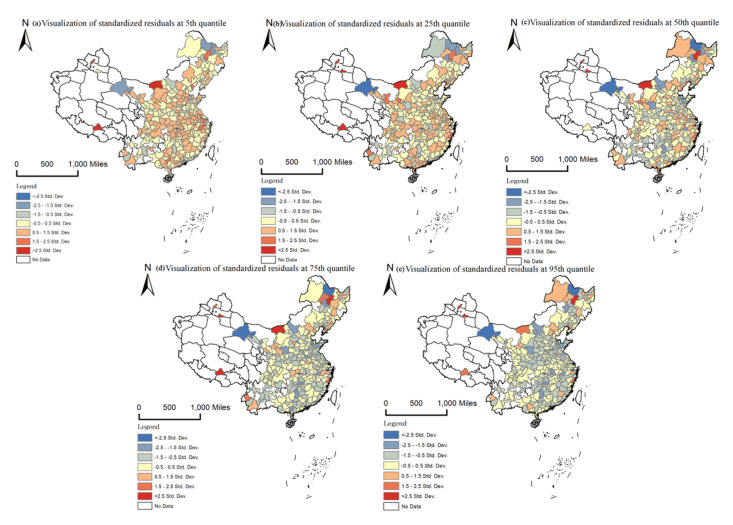
Spatial visualization of standardized residuals of GWQR for 2013. (**a**–**e**) represent the spatial distribution of standardized residuals for the 5th, 25th, 50th, 75th, and 95th quantiles respectively.

For the purpose of revealing non-stationary effects of PM2.5 pollution on urban housing prices at different quantiles, we use the GWQR model to estimate the coefficients. Table 4 lists the descriptive statistics of GWQR coefficient estimates of PM2.5 pollution variable for the five selected quantiles. All *R*^2^ are within a reasonable range, and the standardized residuals (most the absolute value of them are less than 2.5) in Figure 5 also prove that the GWQR models are appropriate.

According to means and medians of GWQR coefficients in Table 4, the trends of PM2.5 coefficients are similar to QR estimates for 2013. The coefficients are dominated by a negative correlation for PM2.5 pollution, and these negative relationships become stronger at most quantiles and especially at the 5th and 95th quantiles. GWQR model coefficients are spatially varying throughout the study area. Following Chen’s method [38], spatial non-stationary effects of the response variable and accompanying predictor variable are assessed by judging whether the interquartile ranges (IQRs) of the localized coefficients are more than twice the standard errors of the global estimates. Table 4 indicates that IQRs of the local estimates of PM2.5 pollution were at least twice the standard error of global estimates in 2013. Our results suggest that spatial relationships between housing prices and PM2.5 pollution indeed vary across the studied prefecture-level cities.

Table 4 reports the GWQR estimates of PM2.5 pollution for 2005 and 2009 to draw comparisons. The relationship between PM2.5 pollution and housing prices was dominated by negative correlations from 2005 to 2013. According to the recorded means and medians, the absolute values of the regression coefficients mostly increase overtime, which is consistent with QR estimates. Moreover, GWQR estimates of most quantiles for 2005 are similar to QR estimates. GWQR estimates are smaller than QR estimates at the high quantile, and GWQR estimates are greater than QR estimates at the low quantile in 2009.

The impacts of PM2.5 pollution on urban housing prices are spatially varying in most quantiles except at the 50th quantile in 2005, and the impact of PM2.5 pollution on urban housing prices shows spatial stability at 5th and 25th quantiles in 2009. This indicates that there are no significant spatial differences in their regression coefficients at the two quantiles.

**Figure 6:**
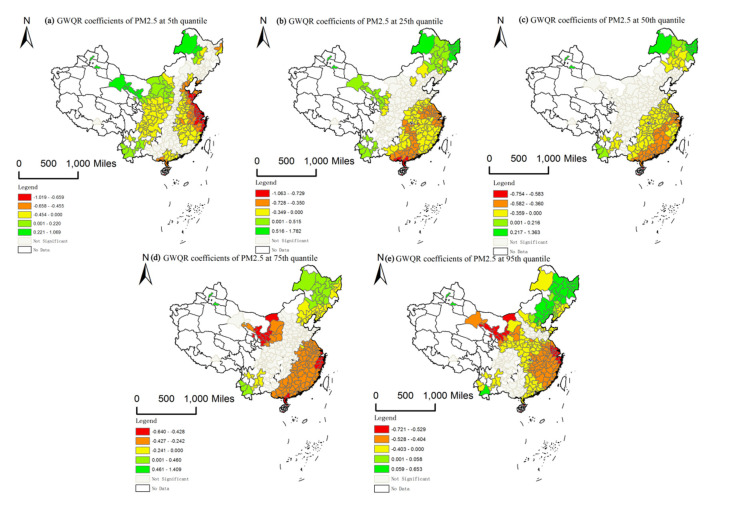
Spatial visualization of significant PM2.5 coefficients of GWQR for 2013. (**a**–**e**) represent the spatial distribution of significant PM2.5 coefficients for the 5th, 25th, 50th, 75th, and 95th quantiles respectively.

**Figure A1:**
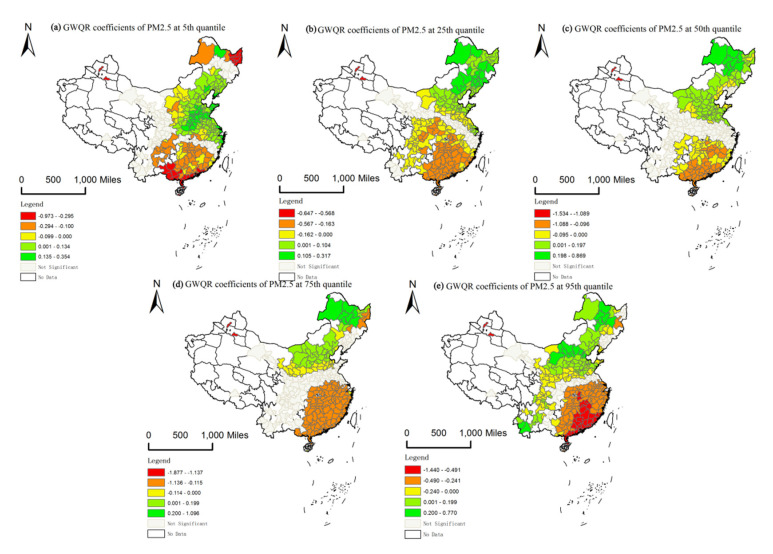
Spatial visualization of significant PM2.5 coefficients of GWQR for 2005. (**a**–**e**) represent the spatial distribution of significant PM2.5 coefficients for the 5th, 25th, 50th, 75th, and 95th quantiles respectively.

**Figure A2:**
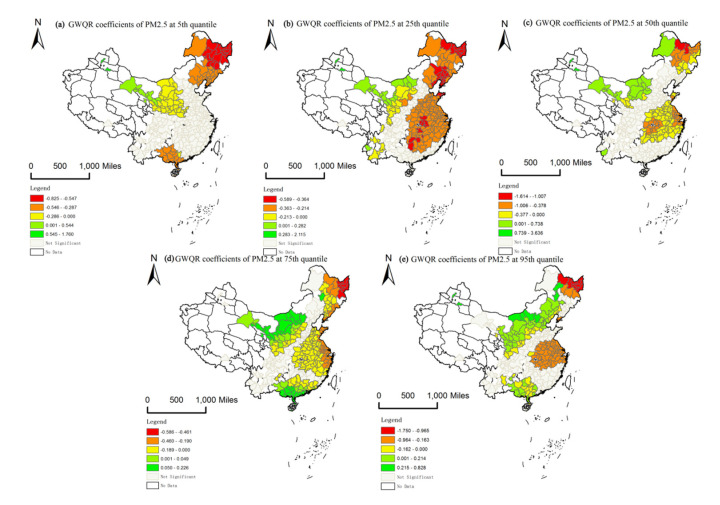
Spatial visualization of significant PM2.5 coefficients of GWQR for 2009. (**a**–**e**) represent the spatial distribution of significant PM2.5 coefficients for the 5th, 25th, 50th, 75th, and 95th quantiles respectively.

Furthermore, model coefficients of each location were calculated by the GWQR method in the study area at specific quantiles. We visualized PM2.5 pollution coefficients using the ArcGIS platform (ESRI, Redlands, CA, USA), which generated spatially varying trends of urban housing prices in response to PM2.5 pollution. Following Chen’s approach [38], we employ model coefficients to construct geographic maps for which local t-test statistically significant (t values exceed +/− 1.96) at the five quantiles (*t* = 0.05, 0.25, 0.50, 0.75, and 0.95) for PM2.5 pollution across the three time series (Figure 6, Figure A1 and Figure A2).

Figure 6 shows estimates and significant areas for PM2.5 pollution for 2013. At most quantiles, a negative correlation is obviously found between PM2.5 pollution and urban housing prices in Eastern China (including Jiangsu, Zhejiang, Shanghai, Anhui, and Fujian), and positive correlations are mainly found in Northeastern China and Yunnan province. Moreover, positive correlations are stronger at higher quantiles. At all quantiles, stronger negative correlations are mainly distributed across the eastern and southern coastal areas, indicating that real estate markets in these areas are very sensitive to PM2.5 pollution. Northwestern and Southwestern China show the opposite relationship between urban housing prices and PM2.5 pollution at the 5th quantile. Specifically, PM2.5 pollution negatively affects urban housing prices in Southwestern China, while estimates of PM2.5 pollution in Northwestern China show a positive correlation but a negative correlation at high quantiles. In addition, areas showing significantly positive and negative correlations expand at the 95th quantile.

This spatial differentiation of influences of PM2.5 pollution on housing prices in northern and southern China is attributable to the combined effects of natural environmental conditions, human activities, and economic development. For example, while coal-fired heating is used in the northern region, it is largely not adopted in southern China, and factors such as terrain and weather patterns create differences in PM2.5 pollution. Meanwhile, China’s economic development pattern mainly presents the characteristics of fast in the south and slow in the north. More developed regions are less likely to adopt unsustainable development modes that pollute the environment and have greater demands for environmental quality in southern China. Therefore, PM2.5 pollution is always negatively correlated with urban housing prices in China’s eastern coastal cities at higher quantiles.

Compared with the southern China, industrial pollution is more serious in northern region. The heavy chemical industry is highly concentrated in Beijing-Tianjin-Hebei urban agglomeration and Northeast China. The coal-based energy utilization mode and the highway transportation mode lead to the high emission of air pollutants in the region. In these areas, the rapid development of urban economy is at the expense of environmental health. It brought economic development and a real estate boom, but it is unsustainable

The pollution level in southwest China is relatively low. Along with China’s rapid urbanization, the inland provinces represented by Yunnan and Guizhou are gradually industrializing through industrial relocation and investment in massive railways and public infrastructure. Therefore, PM2.5 pollution and housing prices in these areas also show positive correlations. Maintaining the balance between economic growth and environmental health is an important issue for policymakers to consider.

Figure A1 and Figure A2 show the GWQR estimates of PM2.5 pollution for 2005 and 2009, respectively. In 2005, impacts of PM2.5 pollution on urban housing prices show regional differentiation between Northern and Southern China at all quantiles in the study area, where most PM2.5 pollution estimates for the northern region are positively correlated with urban housing prices while relationships are mostly negative in the southern region. Spatial distributions changed considerably in 2009 and the area of northern China with a positive correlation greatly decreased. In the study area, a broader range of positive coefficients is found in 2005 than in 2009 and 2013. In the time series, negative correlations gradually dominate. Thus, impacts of PM2.5 pollution on urban housing prices continuously intensified nationwide. Consistency between the three time sections is also observed. At the high quantile, PM2.5 pollution is negatively correlated with housing prices all the time in eastern coastal region.

In summary, we find obvious signs of spatial differentiation in influences of PM2.5 pollution on urban housing prices, and most quantiles show spatial non-stationary characteristics in the three time series. Initially, negative and positive effects are found in northern and southern China, respectively. Then, the area of northern China showing positive effects also steadily decreases. By 2013, only the northeastern region shows positive effects, and negative effects dominate most of the study area.

According to the Environmental Kuznets Curve (EKC) theory, an inverted “U”-shaped curve characterizes the relationship between environmental pollution and economic development [70]. In the initial stage of economic development, the increase in per capita income will lead to the increase of environmental pollution. When the economy develops to a higher level, the environmental pollution problem is alleviated. Early economic growth is usually an extensive growth at the expense of environmental health. With the increase of technological level and environmental awareness, people pay more attention to the coordinated development of the economy and the environment. In China, there are related studies that prove this point [71,72]. It is foreseeable that developed regions will reach the inflection point of the inverted “U”-shaped curve early. Meanwhile, developed areas will have stricter air quality requirements. As a result, air pollution will cause greater losses to housing prices and the economy. They are also willing to pay more marginal prices for clean air.

For the industrial cities that pursue economic development, their production process will produce a lot of pollution. They have achieved rapid economic development at this cost, including housing prices. Therefore, the positive correlation between housing prices and PM2.5 pollution will be formed in the early stage of development. This is particularly evident in the northeast region, which is based on heavy industry. With the transformation of industries, their development will be towards low pollution and low energy consumption. In recent years, some areas will also turn to negative correlations. Therefore, we believe that the spatial non-stationary effects of PM2.5 pollution on housing prices is of great significance for guiding regional development.

### 3.4. Robustness Checks

The common kernel function types of the GWR model include Gaussian kernel function, exponential kernel function, and bi-square kernel function. The bandwidth selection methods include the CV method and Akaike Information Criterion (AIC) method [73,74]. In Zhen’s research, the AIC method is used as the bandwidth selection method of GWQR model [40]. Therefore, exponential kernel function and the AIC method are used to test the robustness of the empirical findings of the GWQR model.

Table A1 and Table A2 in Appendix A report the regression parameters using exponential kernel function and AIC methods, respectively. Our results are well grounded. The five selected quantiles of the GWQR coefficient are mostly non-stationary in the three time sections (Column 9 in Table A1 and Table A2), which is consistent with the results in Table 4. Based on the ‘negative proportion’ column of Table A1 and Table A2, we find that the number of negative correlation increases with the time series, which also confirms that the influences of PM2.5 pollution on housing prices are gradually dominated by negative correlations.

**Figure A3:**
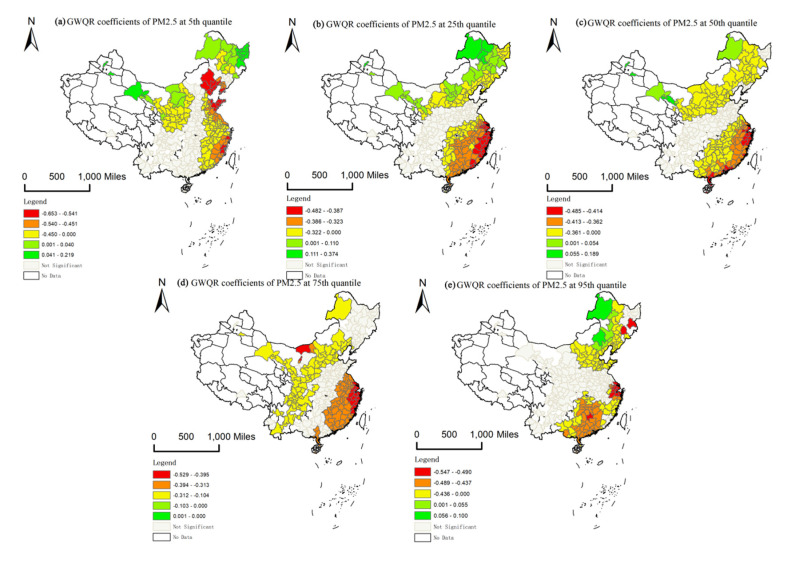
Spatial visualization of significant PM2.5 coefficients of GWQR (exponential kernel function) for 2013. (**a**–**e**) represent the spatial distribution of significant PM2.5 coefficients for the 5th, 25th, 50th, 75th, and 95th quantiles respectively.

**Figure A4:**
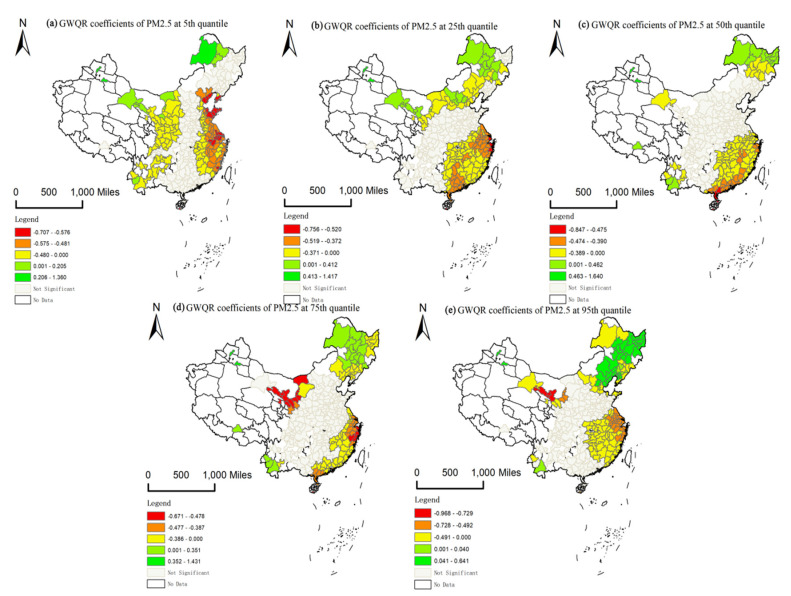
Spatial visualization of significant PM2.5 coefficients of GWQR (AIC method) for 2013. (**a**–**e**) represent the spatial distribution of significant PM2.5 coefficients for the 5th, 25th, 50th, 75th, and 95th quantiles respectively.

Figure A3 and Figure A4 show the spatial visualization of significant PM2.5 coefficients of GWQR using exponential kernel function and AIC method for 2013, respectively. In terms of spatial distribution of PM2.5 coefficients, the negative correlations are concentrated in the eastern and southern coastal areas, while the positive correlations are mainly distributed in the Northeast China, which is consistent with the results in Figure 6. Although the significant areas are slightly different, the main findings are not evident affected. In summary, using the alternative kernel function and bandwidth selection method of the GWQR model supports the empirical findings, and the main results of this study are robust.

## 4. Conclusions

PM2.5 concentrations started to become a concern for Chinese citizens in 2013, and air pollution has aroused wide concern in the public. Despite growing concern over the effects of PM2.5 pollution on urban housing prices, studies have rarely considered asymmetric and spatial non-stationary effects at different housing price levels. To address this gap, this study employs quantile regression and geographically weighted quantile regression models to measure the asymmetric and spatial non-stationary effects of PM2.5 pollution on urban housing prices at different quantiles. Considerable variations are found across different quantiles and spaces, indicating that quantile and geographically weighted quantile regressions can provide highly comprehensive descriptions. This paper offers three main empirical conclusions:

(1) The data distribution patterns of PM2.5 pollution and urban housing prices are similar across the three time series. However, the relationship between urban housing prices and PM2.5 pollution is asymmetric according to our data distribution, and a spatial non-stationary relationship between them may be found by visualizing their spatial characteristics.
(2) Our results of OLS and QR regression models confirm the negative effects of PM2.5 pollution on urban housing prices. Moreover, these negative effects are stronger at higher quantiles, reflecting asymmetric effects between them. Further, the influences of PM2.5 pollution on housing prices increase in our time series. Meanwhile, the range of PM2.5 pollution impacts are expanding.
(3) GWQR models can produce novel and original findings with more data on spatial variations in influences of PM2.5 pollution on urban housing prices. We find spatial relationships to be non-stationary at most quantiles in our three time series, revealing spatial heterogeneous effects of PM2.5 pollution on urban housing prices. In our time series, negative influences of PM2.5 pollution on urban housing prices expand nationwide overtime. Higher priced cities of eastern costal China are always negatively affected by PM2.5 pollution and remain stable in our time series. It should be noted that positive and negative correlations found between PM2.5 pollution and urban housing prices are stronger at higher quantiles.

## 5. Policy Implications and Future Perspectives

Real estate constitutes an important asset for Chinese families and the real estate market is also very speculative. Investment in the housing market is often the consequence of a comprehensive balance of multiple factors (e.g., higher incomes, personal development, and desires for a better living environment). PM2.5 pollution is bound to affect people’s well-being and enthusiasm for investment in real estate. Specifically, enthusiasm for investment in small and medium-sized cities with clean living environments has increased alongside a reduced willingness to live in large cities with seriously negative impacts on health. Local governments must guide housing consumption demand in an orderly and reasonable manner, limit the formation of real estate bubbles in medium- and small-sized cities due to speculation, and actively manage large cities with severe air pollution to enhance their overall appeal.

As air pollution and economic development are different between northern and southern China, environmental regulations must be regionally differentiated. PM2.5 pollution in developed southeast coastal areas causes more loss of housing prices at the higher quantiles. Therefore, a more stringent environmental regulation policy could be formulated in order to control air pollution in these areas. However, it should be noted that the impact of air pollution on adjacent areas shows clustering characteristics. When formulating environmental regulations and policies in a certain area, it is necessary to consider the intensity of environmental regulations in surrounding cities, to avoid the relocation of heavily polluting industries and the formation of ‘polluted paradise’ [75]. Therefore, the urban agglomerations need to promote regional atmospheric linkage management, and advocate the allocation of ecological compensation responsibility of more developed cities [76]. For northern cities, whose economic growth are driven by heavy industry, excessive environmental supervision may hinder green technology innovation, and thus may increase the level of PM2.5 pollution. In view of this, the local government should strengthen the environmental supervision to an appropriate level, gradually eliminate the heavy pollution industries that originally supported economic growth, and further promote the reasonable industrial structure. It is imperative to accelerate the transformation and upgrading of old industrial bases, remediate or sanction plants generating high levels of pollution, and optimize the spatial layout of polluting industries. For all regions, it is very important to improve residents’ awareness of environmental protection [77]. The government needs to advocate the concept of green development and the residents should consciously practice the green lifestyle. Meanwhile, it is necessary to encourage social management of PM2.5 pollution, beautify urban spaces, and enhance efforts to support new energy automobile industries and other emerging sustainable industries for promoting the sustainable economy growth.

Three avenues for further research can be identified. First, the GWQR method can help researchers explore local relationships across conditional distributions of a response variable and thus provide more insight into the real estate market and into environmental regulation. While the present work is based on optimal bandwidth levels as determined by the CV and AIC methods, other methods such as the Bayesian Information Criterion are worth applying to find optimal bandwidths. Second, due to data limitations, this study uses section data for 2005–2013. However, the Chinese government has begun to take into consideration the impacts of PM2.5 pollution on people’s lives and has launched a series of prevention and control measures since 2013. Therefore, the relationship between the real estate market and PM2.5 pollution must be continually monitored. Third, in this work, we analyze asymmetric and spatial non-stationary effects of PM2.5 pollution on urban housing prices at different quantiles. However, trade-off mechanisms of PM2.5 pollution and other favorable factors are complex for different quantiles and regions. Future studies need to pay particular attention to the trade-off mechanism between air pollution and other social and economic factors, and then provide more targeted references for formulating regional real estate development and environmental protection policies.

## Figures and Tables

**Table 1:** Variable descriptions and descriptive statistics for 2013.

Variable Type	Variables (Abbreviation)	Variable Definition	Mean	Std. Deviation	Min.	Max.	*n*
Dependent variable	Housing prices (HP)	Average sale price of newly-built homes (RMB/m^2^)	4977.537	2688.35	2435.897	24,401.97	286
Core independent variable	Particulate matter 2.5 pollution (PM2.5)	Fine particulate matter 2.5 concentrations (µg/m^3^)	38.3048	17.7262	2.8446	86.4799	286
Other air pollution variables	SO_2_ pollution (SO_2_)	SO_2_ emissions (10 thousand tons)	5.3650	5.7516	0.0003	49.4415	286
	Soot pollution (Soot)	Soot emissions (10 thousand tons)	4.3443	18.9197	0.0221	315.3822	286
Demographic and socioeconomic variables	Population density (PDen)	Population density (persons/km^2^)	433.1137	338.1286	5.71	2616.23	286
	GDP per capita (PGDP)	GDP per capita (RMB)	51,597.98	48,319.52	8407	467749	286
	Wage (Wage)	Annual wage per worker (RMB)	44,869.77	9648.529	24,786.31	93,996.77	286
	Industry Structure (Ind_Str)	Ratio of tertiary industry to secondary industry	0.7817	0.4069	0.2072	3.4431	286
Urban public facilities variables	Buses_Per (PBu)	Number of buses (units/10 thousand persons)	8.2688	7.3635	0.59	98.53	286
	Books_Per (PBo)	Number of books (volumes/100 persons)	58.7365	86.5789	1.74	920.77	286
	Doctor_Per (PDoc)	Number of Doctors (persons/10 thousand persons)	21.3844	10.3089	2.1728	81.6763	286
	Road_Per (PRoad)	The areas of road (km^2^/person)	13.9865	26.9049	1.04	442.95	286
	Internet(Int)	Number of households connecting Internet (10 thousand)	73.0315	91.6900	5	766	286
	Teacher_Per (PTea)	Number of university teachers (persons/10 thousand people)	10.0134	13.7198	0.0082	81.6855	286

Notes: The descriptive statistics of all variables are based on 286 sample cities in 2013.

**Table 2:** Ordinary least squares (OLS) and quantile regression results for 2013.

Variables	OLS (1)	5th (2)	25th (3)	50th (4)	75th (5)	95th (6)
PM2.5	−0.1986 *** (0.0418)	−0.0359 (0.1007)	−0.1682 *** (0.0505)	−0.1983 *** (0.0353)	−0.2697 *** (0.0665)	−0.2480 ** (0.0997)
SO_2_	−0.0279 ** (0.0112)	−0.0258 (0.0178)	−0.0180 (0.0145)	−0.0231 (0.0140)	−0.0245 (0.0194)	−0.0207 (0.0265)
Soot	−0.0245 ** (0.0120)	−0.0007 (0.0153)	−0.0245 (0.0169)	−0.0271 ** (0.0138)	−0.0304 * (0.0174)	−0.0341 (0.0293)
PDen	0.1585 *** (0.0244)	0.0908 (0.0616)	0.1666 *** (0.0261)	0.1756 *** (0.0239)	0.1594 *** (0.0363)	0.1094 * (0.0557)
PGDP	0.0892 *** (0.0331)	0.0795 (0.0727)	0.0755 (0.0474)	0.0693 (0.0523)	0.1308** (0.0526)	0.1362 * (0.0695)
Wage	0.6306 *** (0.0851)	0.5162 *** (0.1074)	0.6107 *** (0.1030)	0.7236 *** (0.1149)	0.7137 *** (0.1321)	0.8288 *** (0.0957)
Ind_Str	0.1398 *** (0.0345)	0.1268 ** (0.0518)	0.1496 *** (0.0461)	0.0822 * (0.0468)	0.1185 ** (0.0482)	0.1866 *** (0.0567)
PBu	0.0094 (0.0231)	−0.0099 (0.0380)	−0.0180 (0.0165)	0.0234 (0.0263)	0.0158 (0.0336)	−0.0102 (0.0396)
PBo	0.0588 *** (0.0204)	0.0558 (0.0557)	0.0766 *** (0.0286)	0.0863 *** (0.0253)	0.0372 (0.0235)	0.0568 *** (0.0214)
PDoc	−0.0159 (0.0344)	−0.0367 (0.0532)	−0.0282 (0.0433)	0.0028 (0.0409)	−0.0405 (0.0575)	−0.0397 (0.0673)
PRoad	−0.0291 (0.0327)	−0.0051 (0.0424)	−0.0298 (0.0336)	−0.0349 (0.0389)	−0.0196 (0.0439)	−0.0383 (0.0382)
Int	0.1349 *** (0.0230)	0.1031 ** (0.0427)	0.1255 *** (0.0241)	0.1141 *** (0.0223)	0.1295 ** (0.0281)	0.1487 *** (0.0459)
PTea	−0.0002 (0.0137)	0.0350 (0.0364)	0.0040 (0.0263)	−0.0218 (0.0135)	−0.0051 (0.0198)	0.0245 (0.0206)
Intercept	−0.0094 (0.8411)	0.9769 (1.1835)	0.1231 (1.0084)	−0.9638 (0.9627)	−0.8432 (1.1278)	−1.8423 (1.1440)
*R* ^2^	0.7899	0.3749	0.4459	0.5084	0.6016	0.7211

Notes: *** *p* < 0.01, ** *p* < 0.05, and * *p* < 0.1. Standard errors are reported in parentheses. The *R*^2^ for the quantile regression is the pseudo *R*^2^. OLS (1) reports the OLS regression results, and (2)–(6) report quantile regression results for the 5th, 25th, 50th, 75th, and 95th quantiles respectively.

**Table 3:** OLS and quantile regression results for 2005 and 2009.

Variables	OLS (1)	5th (2)	25th (3)	50th (4)	75th (5)	95th (6)
2005						
PM2.5	−0.0661 (0.0454)	0.0226 (0.1082)	−0.0834 (0.0820)	−0.0270 (0.0636)	−0.1447 *** (0.0558)	−0.1705 (0.1358)
*R* ^2^	0.7571	0.3938	0.4325	0.5136	0.5777	0.6078
2009						
PM2.5	−0.1249 ** (0.0520)	−0.0534 (0.0670)	−0.0925 (0.0802)	−0.0751 (0.0721)	−0.1642 *** (0.0623)	−0.2571 *** (0.0915)
*R* ^2^	0.7538	0.4183	0.4323	0.5019	0.5665	0.6578

Notes: *** *p* < 0.01, and ** *p* < 0.05. Standard errors are reported in parentheses. The *R*^2^ for the quantile regression is the pseudo *R*^2^. Due to space limitations, we only list the coefficients for PM2.5 and *R*^2^. The full regression results are available upon request.

**Table 4:** Summary statistics of geographically weighted quantile regression (GWQR) estimate coefficients of PM2.5 for 2005–2013.

Quantiles	Mean	Median	Min.	Max.	Negative (%)	IQR	Ste.	Status	Residuals	*R* ^2^
2013										
5th	−0.2625	−0.2833	−1.0190	1.1865	247/286	0.3093	0.1007	Non-stationary	30.7664	0.2691
25th	−0.1610	−0.1962	−1.0635	1.7825	243/286	0.2699	0.0505	Non-stationary	24.8881	0.4088
50th	−0.2375	−0.2619	−0.7544	1.3632	268/286	0.1537	0.0353	Non-stationary	14.8876	0.6463
75th	−0.2465	−0.2662	−0.6402	1.4088	265/286	0.1679	0.0665	Non-stationary	17.0657	0.5946
95th	−0.2932	−0.3534	−0.7210	0.6526	246/286	0.2167	0.0997	Non-stationary	28.4360	0.3245
2009										
5th	−0.2272	−0.2211	−0.8249	1.7598	274/286	0.1076	0.0670	Stationary	34.9078	0.3429
25th	−0.2303	−0.2789	−0.5886	2.1146	263/286	0.1376	0.0802	Stationary	21.1357	0.6021
50th	−0.1780	−0.1988	−1.6137	3.6359	256/286	0.2314	0.0721	Non-stationary	19.6357	0.6304
75th	−0.0844	−0.0928	−0.5858	0.4919	224/286	0.1351	0.0623	Non-stationary	22.3391	0.5795
95th	−0.1338	−0.1046	−1.7495	0.8277	220/286	0.2200	0.0915	Non-stationary	28.3279	0.4668
2005										
5th	−0.0313	−0.0179	−0.9725	0.5687	150/286	0.2276	0.1082	Non-stationary	39.3096	0.2048
25th	−0.0716	−0.0841	−0.6469	0.3168	200/286	0.1894	0.0820	Non-stationary	18.1943	0.6320
50th	−0.0241	−0.0309	−1.5339	0.8694	182/286	0.0982	0.0636	Stationary	16.9186	0.6578
75th	−0.1088	−0.1260	−1.8769	1.0960	247/286	0.1354	0.0558	Non-stationary	15.2975	0.6906
95th	−0.1820	−0.1651	−1.4802	0.7696	186/286	0.3599	0.1358	Non-stationary	40.8241	0.1742

Notes: IQR values are computed by the estimates of the 75th of the GWQR model minus the estimates of the 25th. Ste.—the standard error of global estimates derived with a traditional QR.

## Appendix A

**Table A1:** Summary statistics of GWQR (exponential kernel function) estimate coefficients of PM2.5 for 2005–2013.

Quantiles	Mean	Median	Min.	Max.	Negative (%)	IQR	Ste.	Status	Residuals	*R* ^2^
2013										
5th	−0.2703	−0.2706	−0.6526	0.2190	263/286	0.2587	0.1007	Non-stationary	19.1201	0.5458
25th	−0.2061	−0.2303	−0.4822	0.3743	257/286	0.2282	0.0505	Non-stationary	12.5511	0.7018
50th	−0.2507	−0.2359	−0.4851	0.1890	279/286	0.1647	0.0353	Non-stationary	8.8104	0.7907
75th	−0.2806	−0.2912	−0.5291	−0.1041	286/286	0.1123	0.0665	Stationary	11.7863	0.7200
95th	−0.3412	−0.3807	−0.5471	0.1001	278/286	0.1258	0.0997	Stationary	18.9354	0.5502
2009										
5th	−0.2205	−0.2487	−0.5534	0.8631	266/286	0.1310	0.0670	Stationary	34.5803	0.3491
25th	−0.2303	−0.2789	−0.5886	2.1146	263/286	0.1376	0.0802	Stationary	23.9320	0.5495
50th	−0.1780	−0.1988	−1.6137	3.6359	256/286	0.2314	0.0721	Non-stationary	23.0096	0.5669
75th	−0.0844	−0.0928	−0.5858	0.4919	224/286	0.1351	0.0623	Non-stationary	19.3198	0.6363
95th	−0.1338	−0.1046	−1.7495	0.8277	220/286	0.2200	0.0915	Non-stationary	26.7136	0.4972
2005										
5th	−0.0037	−0.0276	−0.6137	0.5110	156/286	0.2594	0.1082	Non-stationary	33.0169	0.3321
25th	−0.0684	−0.0755	−0.3471	0.2132	212/286	0.1544	0.0820	Stationary	16.6069	0.6641
50th	−0.0264	−0.0291	−0.2241	0.5743	195/286	0.1057	0.0636	Stationary	13.8040	0.7208
75th	−0.0903	−0.1142	−0.4126	0.7253	234/286	0.1341	0.0558	Non-stationary	17.3223	0.6496
95th	−0.1820	−0.1651	−1.4802	0.7696	213/286	0.3599	0.1358	Non-stationary	36.8610	0.2544

Notes: IQR values are computed by the estimates of the 75th of the GWQR model minus the estimates of the 25th. Ste.—the standard error of global estimates derived with a traditional QR.

**Table A2:** Summary statistics of GWQR (AIC method) estimate coefficients of PM2.5 for 2005–2013.

Quantiles	Mean	Median	Min.	Max.	Negative (%)	IQR	Ste.	Status	Residuals	*R* ^2^
2013										
5th	−0.2842	−0.2776	−0.7071	1.3603	273/286	0.2849	0.1007	Non-stationary	20.3452	0.5167
25th	−0.2014	−0.2118	−0.7562	1.4173	264/286	0.2367	0.0505	Non-stationary	20.7411	0.5073
50th	−0.2376	−0.2571	−0.8474	1.6399	272/286	0.1572	0.0353	Non-stationary	15.0992	0.6413
75th	−0.2462	−0.2612	−0.6709	1.4310	265/286	0.1504	0.0665	Non-stationary	20.5737	0.5113
95th	−0.2953	−0.3516	−0.9676	0.6413	250/286	0.2111	0.0997	Non-stationary	34.4835	0.1808
2009										
5th	−0.2159	−0.2437	−0.6651	2.2310	267/286	0.1225	0.0670	Stationary	32.8354	0.3819
25th	−0.2268	−0.2778	−0.5621	2.3344	262/286	0.1406	0.0802	Stationary	26.1222	0.5083
50th	−0.1714	−0.1869	−1.1915	3.6993	260/286	0.2083	0.0721	Non-stationary	31.2354	0.4120
75th	−0.0853	−0.0991	−0.6407	1.4393	224/286	0.1410	0.0623	Non-stationary	33.3338	0.3725
95th	−0.1338	−0.1046	−1.7495	0.8277	220/286	0.2200	0.0915	Non-stationary	32.5460	0.3874
2005										
5th	−0.0395	−0.0376	−0.9698	0.5338	161/286	0.2396	0.1082	Non-stationary	39.3096	0.2048
25th	−0.0879	−0.0807	−0.8639	0.7156	208/286	0.1998	0.0820	Non-stationary	26.6307	0.4613
50th	−0.0557	−0.0421	−0.6537	1.3645	184/286	0.2326	0.0636	Non-stationary	13.4764	0.7274
75th	−0.0878	−0.1031	−0.7358	1.2812	221/286	0.1950	0.0558	Non-stationary	15.2975	0.6906
95th	−0.1842	−0.1641	−1.4403	0.7081	215/286	0.3815	0.1358	Non-stationary	44.1466	0.1070

Notes: IQR values are computed by the estimates of the 75th of the GWQR model minus the estimates of the 25th. Ste.—the standard error of global estimates derived with a traditional QR.
